# Exploring the Psychometric Properties of the Questionnaire on Family Members Adapting to an Older Adult’s Admission to a Nursing Home (CAFIAR-15) in a Colombian Sample

**DOI:** 10.3390/bs12010004

**Published:** 2021-12-23

**Authors:** Marta Martín-Carbonell, Antonio Riquelme-Marín, Martha Fernández-Daza, Juan Manuel Ortigosa-Quiles, Inmaculada Méndez-Mateo

**Affiliations:** 1 Psychology Department, Cooperative University of Colombia, Troncal del Caribe S/N, Santa Marta 47002, Colombia; martha.fernandezd@campusucc.edu.co; 2 Department of Personality, Assessment and Psychological Treatments, University of Murcia, 30100 Murcia, Spain; riquelme@um.es (A.R.-M.); ortigosa@um.es (J.M.O.-Q.); 3 Department of Evolutionary and Educational Psychology, University of Murcia, 30100 Murcia, Spain; inmamendez@um.es

**Keywords:** seniors, questionnaire development, family adjustment, psychometric properties, nursing home, inequalities, social determinants

## Abstract

Institutionalization to a nursing home can be one of the most significant and traumatic events in a senior’s life, and for their family. For this reason, it is especially important to have validated instruments that evaluate the family member’s adaptation to admitting the senior to a nursing home. The study included 139 family members recruited equally in two types of institutions (low-income nursing home (LINH) vs. high-income nursing home (HINH)). A sociodemographic questionnaire with questions to study antecedents and conditions for care and the Questionnaire for Admitting an Older Adult to a Nursing Home (CAFIAR-15) were used. Examining the communalities indicated that four of the five items in factor 3 presented communalities lower than 0.30 and differences in the factorial structure of the CAFIAR-15 were found. There were differences in the antecedents and conditions for care between the relatives of the older adults at LINH and HINH. Cultural differences and differences between LINH and HINH may be the basis for flaws in the conceptual validity of the CAFIAR-15 in the Colombian sample.

## 1. Introduction

Being institutionalized in a nursing home can be one of the most significant and traumatic events for seniors and their families [1]. In Colombian culture, families prefer to take care of seniors at home. However, there is an accelerated aging of the population in conditions of poverty; economic, social, and gender inequalities; and inequities in accessing health services. This means that a growing number of seniors need support and care that their families cannot provide, and they cannot manage by themselves. Therefore, families increasingly are resorting to admitting frail, dependent seniors to nursing homes to provide care [2,3].

In Colombia, long-stay institutions that house and care for seniors are called social protection centers or nursing homes. In 2020, 1216 social protection centers were registered with the Social Promotion Office of the Ministry of Health; these centers serve 35,438 seniors. It is estimated that there are about 500 unregistered institutions [4]. Currently, residential centers for seniors are centers for the permanent or temporary shelter of seniors, where accommodation, social well-being, and comprehensive care services are offered to seniors [5]; however, it has not always been this way. They arose and developed initially as asylums, and they continue to play this role. In the Colombian context, institutionalization is a response to the multiple deficiencies experienced by populations that live in conditions of poverty and extreme poverty [6] and corresponds to 1% of the total population [7].

Although the information on nursing homes in Colombia is scarce, currently, 38% are non-profit institutions and 62% are small for-profit enterprises, which operate on limited financial resources and are an option to provide relief for families tasked with caring for seniors with severe disabilities [8]. In the last few decades of the past century, various financing methods were used by people who have the means to cover living expenses. This led to the stratification of senior care, ranging from the level of social assistance to the category of pensioners. This modality includes high-cost institutions that offer physical and environmental conditions with a high level of comfort and even luxury.

It is now considered essential for staff working in nursing homes to consider the needs of family members and the challenges they face during institutionalization of seniors [9]. Research has been limited in this regard; however, the importance of families accepting and adapting to a senior’s admission to a home is recognized, both for their own well-being [10,11] and that of the senior’s [12,13], as well as for the proper functioning of the institution [14,15].

Rosenthal and Dawson [16] studied the adaptation process of wives when their spouses with advanced dementia are institutionalized. Butcher et al. [17] studied family members’ processes of deciding to admit a senior with dependency issues to a nursing home. Bowers [18] established a typology of family care. Nolan et al. [19] proposed a typology of seniors admitted to nursing homes. Davies [20] studied the potential usefulness of Meleis’s Transition Model to understand family members’ experiences. In the aforementioned models, the consensus is that admitting seniors to nursing homes is a significant life event with an emotional cost for both seniors and their families. The extant research indicates that a family member’s acceptance and adaptation to a senior’s admission can be interpreted as a process in which negative valence experiences and feelings exist (e.g., feelings of guilt, social stigma, nostalgia for the senior, and concern for the quality of care they will receive) along with feelings of positive valence (e.g., relief of the burden and the opportunity to regain their activities and lifestyle prior to seniors’ deteriorating health).

Although there are multiple instruments for evaluating family caregivers, they have been designed for people who care for seniors in their typical environment and are focused on the perception of overloading the family caregiver [20], an aspect that is substantially modified after the senior moves to a nursing home [21]. The evaluation with standardized tools of the adaptation of the family member is practically absent in the literature. Only two studies have been found: one by Rosén et al. [22] who evaluated quality of life using the World Health Organization’s quality-of-life self-assessment instrument (WHOQOL) in a sample of 254 family members in Sweden, and another work by Pérz-Dorado et al. [23], who applied the “Zarit-caregiver burden scale” to a sample of 20 elderly relatives from a nursing home in Seville. However, quality of life is a broader concept than the adaptation of the family members, although they are interrelated and in the Spanish study with Zarit they found that, according to family members, there was no burden since the care for the elderly was in the hands of the nursing home staff; hence this questionnaire is of little use.

Recently, our team proposed a definition of the construct “family members’ adaptation to a senior’s admission to a home” and a questionnaire to assess this topic, whose psychometric properties were studied in a Spanish sample [24]. The process used to define the construct was described in the aforementioned article and was used to construct the following definition: “a multidimensional construct referring to how the interviewee feels at a given moment in his or her personal adaptation process to a senior’s admission to a nursing home”. This adaptation is understood to be a complex process in which contradictory experiences and feelings of relief, guilt, sadness, nostalgia, and concern for seniors’ well-being can occur. This process occurs with greater or lesser intensity and duration in close relatives, particularly in the main caregiver, in the event that prior to admission, a member of the family had played that role. A relative is considered to be any person, regardless of the degree of kinship, who, prior to the senior’s admission to the nursing home, has had some connection with his or her care and continues to visit the senior after admission.

Based on this conceptual definition, an instrument (CAFIAR-15) was proposed whose psychometric properties were initially evaluated in a Spanish sample of 134 family members of seniors admitted to nursing homes [24]. Using exploratory factor analysis (EFA), a study was carried out with the principal axes vectorization method in a pool of 19 items, it was possible to obtain a 15-item instrument with three factors: factor 1 (unease due to admitting an older adult to a nursing home), factor 2 (relief), and factor 3 (nostalgia and concern for the older adult), in addition to a general adjustment index, with a Cronbach’s alpha of 0.74. The general adjustment index and the subscales that demonstrate poor adjustment were significantly correlated with depression and a worse health self-assessment, while the relief subscale, which indicates better adjustment, was significantly correlated with well-being and a positive health self-assessment.

### Research Purpose

This article assesses whether the CAFIAR-15 is a valid instrument to study family members’ adaptation to a senior’s admission to a nursing home in Colombia, taking into account the recommendations of the International Testing Commission (ITC) [25]. As mentioned above, in our country there are various financing modalities for geriatric homes, with subsequent differences in the socioeconomic level of the clients and their families. There are also cultural differences that can affect the way questionnaires are understood and answered [26]

To our knowledge, there is no instrument that evaluates family members’ adaptation in a quick, valid, reliable way, which is necessary for this area of knowledge to be developed.

It should be noted that the CAFIAR is an instrument under construction, which has only been explored in a limited sample of older adults in Spain, thus we consider that the value of internal consistency reported by the authors could be considered acceptable, taking into account the recommendation from [27] that values greater than 0.7 can be accepted when a new measure is being developed.

The relevance of exploring the validity of CAFIAR-15 in our setting is aligned with the fact that there are few theoretical models proposed to understand the family adaptation process [9,18,19]. In this sense, it should be noted that the construct “adaptation of the family member to the institutionalization of the elderly” itself needs to be perfected, taking into account that there may be important differences in the way in which family members adapt to this situation, as it has been pointed out by research comparing Hispanics with people from cultures such as Anglo-Saxon and African-American [28,29,30,31]. These authors agree that Hispanics are more reluctant to admit an elderly person with dementia to a geriatric institution

On the other hand, a limitation of the few conceptual proposals regarding the adaptation of the family member to institutionalization outlined above [16,17,18,19,20] is that they have been developed from qualitative studies with small samples; they are contextualized in Western culture, in economically developed countries, and performed in the late twentieth century. This may lead to certain characteristics of the ones described in said proposals not adjusting to the current Colombian reality.

For these reasons, in this study, we set out to explore some psychometric properties of CAFIAR-15 in the Colombian population, specifically we wanted to obtain information on internal consistency and explore its factorial structure in a Colombian sample of relatives of institutionalized older adults.

## 2. Materials and Methods

### 2.1. Participants

The sample, comprising 139 family members, was selected on the basis of the feasibility of obtaining information. The inclusion criteria were as follows: (1) nursing homes located in Colombia (Caribbean region) that agreed to collaborate with the researchers by providing contact information for the residents’ family members; (2) persons who gave informed consent to participate in the study, and (3) any person, regardless of the degree of kinship who, prior to the senior’s admission to the nursing home, had had some connection with their care and continues to visit the senior after admission.

The sample was collected from six nursing homes in four cities from the Colombian Caribbean, on the country’s northern coast. Two of the homes, located in the district’s capital, are for people with high economic resources who can finance the admission of the seniors. We defined these two centers as high-income nursing homes (HINH) to facilitate communication. They contributed 70 participants (50.4% of the total sample).

The remaining four homes, funded by the Catholic Church, charitable organizations, or local governments, accept people without means or who pay low-cost fees. We defined these as low-income nursing homes (LINH), and 69 participants (49.6% of the total sample) were recruited from them.

### 2.2. Instruments

The family members’ adaptation to a senior’s admission to a nursing home (CAFIAR-15) scale [24] consists of 15 items with three factors to evaluate feelings of negative and positive valence that family members of seniors admitted to nursing homes may experience. The questions have five response options on a Likert scale to assess the presence and intensity of these feelings at the current moment, regarding the fact that their relative is admitted to a home (i.e., “None”, “A little”, “Moderately”, “A lot” and “Completely”). It consists of three factors or subscales, each with five items: factor 1, which assesses emotional distress related to feelings of guilt over the senior’s admission to the residence; factor 2, which assesses relief and the perception of regaining one’s social life; and factor 3, which explores feelings of nostalgia and concern for the senior. Further, a general adaptation index was obtained by adding factors 1 and 3, which assess feelings of negative valence, and subtracting from this sum the score obtained in factor 2, which assesses feelings of positive valence. The instrument has shown a Cronbach’s alpha reliability of 0.74 in the Spanish population [24].

Additionally, a structured interview was conducted to obtain information on the sociodemographic characteristics of family members (e.g., sex, age, marital status, employment status, and education level), history of care prior to admission (e.g., relationship with the senior admitted to the home, role played in caring for the senior before they were admitted to the nursing home, previous living situation, and reasons for admission), and current conditions for care (e.g., distance from home to the nursing home, frequency of visits, and satisfaction of visits with the senior).

### 2.3. Procedure

The data come from a broader research study. Only the data related to evaluating the CAFIAR-15 in Colombia were included in this report.

To assess whether the items were written understandable for Colombians, the questionnaire was evaluated by judges. The judges were two psychologists with experience in validating psychological instruments and three psychology students in their final year, who were asked to independently evaluate if the items presented any comprehension difficulties for the Colombian population. Everyone was asked to rate their understanding of each item, using a 3-point scale depending on whether the item was clear and understandable (3), difficult to understand (2), or incomprehensible (1). There was 100% agreement between the judges in rating all items with the highest score.

CAFIAR-15 was applied during an interview with individual family members, carried out in nursing homes at the time of the visit or in seniors’ family members’ homes. The interviewers were psychology students in their final year who were trained in data collection (previously trained by the principal investigators). The interviews were conducted in 2019.

### 2.4. Data Analysis

The descriptive statistics of the continuous variables were obtained; the frequency and percentage distributions of the categorical variables were studied; and the chi-square statistic and student t-test were used to analyze the significance of the differences in qualitative and quantitative variables, respectively, to characterize the samples.

The descriptive statistics of the items and the percentage of responses were studied. Cronbach’s alpha coefficient was calculated for each subscale; and item-scale correlations were reviewed. Following Riquelme et al.’s [24] study, items that presented z-scores outside the range of ±3, asymmetry and either kurtosis greater than −2 and +2, factor saturations less than 0.30, or both, were considered.

To evaluate the structure of the scale in the Colombian sample, the same procedure was used as in the Spanish study [24]—exploratory factor analysis (EFA) with the principal axis factoring method and oblimin rotation. We choose EFA because we were interested in identifying the number and composition of common factors (latent variables) necessary to explain the common variance of the set of items, since it is the first time that this instrument has been applied in the Colombian population. The criterion for determining the number of factors to extract was theoretical, considering the results found in the original study, as it is recommended by different authors [32,33]. Subsequently, three factors were extracted.

The analyses were carried out using the Statistical Package of Social Sciences, version 26 (Armonk, NY: IBM Corp) [34], licensed by Universidad Cooperativa de Colombia.

### 2.5. Ethics Approval

This research study is based on the ethical principles and recommendations of the Declaration of Helsinki [35]. It adheres to the general principle that the concern for the well-being of participants takes precedence over scientific interests. Law 1090 of 2006 of Colombia, which regulates the practice of the psychology profession and establishes the Code of Ethics and Bioethics and other provisions, was also considered, specifically aspects related to participation in the study because it was confidential, anonymous, and voluntary. Additionally, provisions for participant well-being and research with human participants were considered. The study was approved by the Ethics Committee of the Universidad Cooperativa de Colombia.

## 3. Results

### 3.1. Sociodemographic Characteristics and Antecedents of Care

First, the sociodemographic characteristics of the sample are briefly described, as well as the past and current conditions in caring for seniors that the interviewed relatives reported.

The participants were between the ages of 50 and 80 years (*M* = 51.78; *SD* = 12.65); 69.8% were women. With regard to employment situation, 59.7% worked outside the home, 23.3% were homemakers, and 17.1% were unemployed. With regard to education level, 88.5% had a medium-high education level (61% with a higher level); 8.1% had primary education levels; and 1.5% were uneducated. With regard to relationship status, 58.3% lived with a partner in a stable relationship. No significant differences were found between the family members connected to HINH and those from the LINH in any of these variables (Table 1).

With regard to family connection with the senior resident, daughters (44.5%) were the most common, 31.1% were related in other ways (the most common were nieces and nephews, 17.6%; daughters- or sons-in-law, 4.2%; and siblings at 5.9%), and 24.4% without kinship ties (neighbors, friends, or informal caregivers). In this variable, significant differences were found attributable to the type of center (X^2^ = 20.25, *gl* = 2, *p* = 0.000) because 68% of the family members of HINH seniors were sons or daughters and 22% had other types of family connections. Among the interviewed relatives of the seniors who were in LINH, only 27.5% were sons or daughters and mostly comprised people without family relationships (34.8%) or other types of kinship, such as nieces and nephews, daughters- or sons-in-law, or siblings (37.7%).

With regard to the role played in caring for seniors prior to their admission to a home, 43.2% of the interviewees stated that they were the main caregiver, 41.7% had performed caregiving tasks, and 15.1% had not been the main caregivers. Furthermore, for this variable, statistically significant differences were found between HINH and LINH (X^2^ = 11.30, *gl* = 2, *p* = 0.004) most frequently among the family members of HINH seniors who were primary caregivers (52.9%) and people who shared caregiving tasks (41.4%). Further, 81% of the participants who were not caregivers were in the LINH, constituting 24.6% of the family members recruited in the LINH. In this subgroup of family members from the LINH, only 33% had been main caregivers; 42% had cared for seniors sporadically before they were admitted to the home.

**Figure 1:**
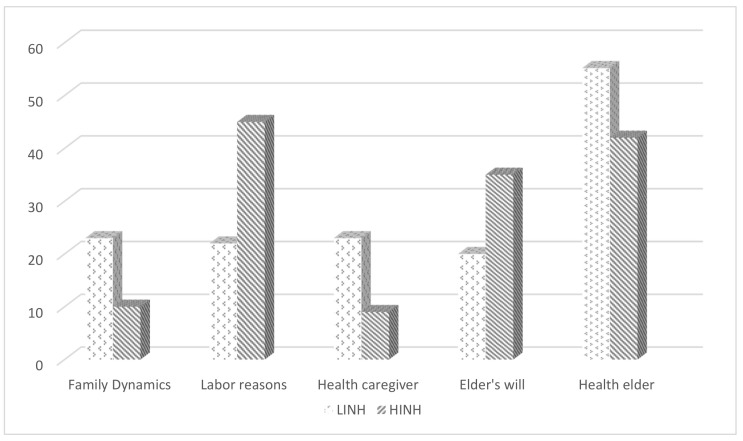
Frequency distribution of responses on reasons for admission according to types of nursing homes (low vs. high resources).

With regard to the reasons that family members considered when deciding to admit seniors to nursing homes (Figure 1), seniors’ worsening health was the most mentioned reason without statistically significant differences attributable to the type of center; work was mentioned significantly more by the family members from the LINH (X^2^ = 7.82 *gl* = 1, *p* = 0.001). Although less mentioned, the affectation of the caregiver’s health was significantly more influential for family members of HINH seniors (X^2^ = 5.42 *gl* = 1, *p* = 0.02), as well as interference in family dynamics.

### 3.2. Psychometric Properties of CAFIAR in the Colombian Sample

Second, the psychometric properties of the CAFIAR-15 are described in the studied sample (Table 2): the response percentage of the items was high. No item was outside the established parameters of skewness, kurtosis, and z-scores; however, 60% of the items presented item-scale correlation values below 0.2.

Cronbach’s alpha values were very low for the subscales “Feelings of guilt” (0.395) and “Nostalgia” (0.341), as well as for the total scale (0.41); while for factor 2 “Relief” can be considered acceptable (0.734).

With regard to the EFA, the Kaiser–Meyer–Olkin measure of sampling adequacy was 0.629 and Bartlett’s test of sphericity was significant for *p* < 0.001, thus proving the suitability of the matrix for factor analysis. However, assessing communalities indicated that four of the five items in factor 3 presented communalities lower than 0.30 (“I am concerned that the nursing home staff does not take good care of my family member” = 0.168; “I think my family member’s health is going to get worse in the short term” = 0.187; “I feel that another person cannot take care of my family member” = 0.088) and two from factor 1 (“I miss my family member” = 0.011 and “I want my family member to come home” = 0.115). Furthermore, the EFA was not able to replicate the factorial structure that was found in the Spanish sample. While the “relief/regaining social life” factor was maintained, the negative valence factors of “guilt” and “nostalgia” dissolved into groups with no theoretical meaning (Table 3). In fact, the items related to nostalgia for the family member that in the Spanish sample were in factor 3 (“I miss my family member” and “I feel that another person cannot take care of my family member”) were not included in any factor.

## 4. Discussion

To advance research and to implement interventions with impact indicators, it is necessary to have measurement instruments that are valid and reliable. As we already mentioned, thus far they have not been generated, so the CAFIAR-15 is a starting point.

A major step in developing and evaluating an instrument to study a multidimensional construct is to theoretically and empirically identify the underlying structure. In psychometric research, the term construct has been linked to validity, emphasizing the importance of an instrument “actually measuring what it claims to measure” [35]. To do so, a characterization of the phenomenon to be evaluated through the theoretical construct is needed, which can be made up of a series of interrelated dimensions.

Our results question the validity of the CAFIAR-15 construct for the Colombian context and indicate the need to identify items and dimensions that can truly be considered valid markers of the construct of a family member’s adaptation to a senior’s admission to a nursing home in Latin America. While an acceptable alpha coefficient (0.70) was found in the Spanish sample and a consistent dimensional approximation had been achieved with the theoretical assumptions of the family member’s adaptation to a senior’s admission to a nursing home construct, in the Colombian one, the Cronbach’s alpha value for the subscales feelings of guilt and nostalgia, were less than 0.50, which is unacceptable [27]

Further, 60% of the items presented item-scale correlation values lower than 0.2, which indicates that they should be discarded or reformulated [32,36,37]. It was not possible to replicate the factorial structure; in fact, the items showing feelings of nostalgia and concern for family members had no explanatory value in the Colombian sample.

These deficiencies found in the construct validity of the CAFIAR-15 in the Colombian sample may have several explanations that are not mutually exclusive.

A possible explanation could be that the Colombian sample is more heterogeneous because it comes from two types of nursing homes (HINH vs. LINH). There are important differences between these types of institutions: while all the LINH complied with the minimum requirements required by MINSALUD Colombia [38] from a structural point of view, they did not have optimal conditions for people with disabilities (e.g., ramps, adapted bedrooms, etc.). Further, some deficiencies in hygiene were noted, and a lack of resources to maintain the infrastructure was evident. In LINH, human talent is a minimum requirement of MINSALUD according to Law 1315 of 2009, which establishes the minimum conditions that dignify a senior’s stay in protection centers and care institutions. Law 1315 establishes that “…The technical direction of these establishments will be assumed by health personnel and/or the social sciences area (technological or professional level), preferably with training in gerontology, psychology, social work, physiotherapy, speech therapy…”. In addition to previously mentioned officials, they must employ the following staff: nutritionists, occupational therapists, cleaning staff, food handlers, and assistants. The law also adds that “The staff must be increased proportionally in relation to the number of beds and the degree of dependence of the residents…”. However, most LINHs are not fully staffed [39]. Further, to provide health and recreation services, they rely on nearby schools, universities, and institutions that provide access to specialized personnel (e.g., doctors and psychologists.) as an opportunity for academic or training experience.

Unlike LINHs, HINLs meet the structural and human talent requirements that are considered necessary for such institutions. Some of the seniors have private rooms, a private caregiver or nurse, internet access, and cell phones. In this sense, the family members in the Spanish sample were more similar to the family members captured in the Colombian HINLs, since the Spanish sample was more homogeneous because all cases came from three residences in the Region of Murcia, located near the capital, which housed the seniors whose families paid for their care.

Unfortunately, the small size of these subgroups in our sample (HINH vs. LINH) did not allow us to study the psychometric properties of the scale based on the type of center, but it is reasonable to assume that in our environment, the heterogeneity of the characteristics of the family members could determine that other items are better markers or that the instrument may have another dimensional structure.

In this sense, our study showed important differences between family members of seniors living in HINH and those living in LINH, first, in the type of kinship of the family members interviewed. There was a high percentage of people without kinship ties among the LINH interviewees. This circumstance leads us to point out the appropriateness of considering the differential characteristics linked to LINH and HINH in relation to adapting to a senior’s admission to a nursing home as a different reality from that studied in the Spanish context. In fact, anecdotally, we can report that in LINHs, a large number of seniors were not visited by their close relatives; however, we did not collect the data for statistical analysis. Our results also coincide with the findings of other Colombian authors that residents in LINH have less family support than those admitted to private nursing homes, especially from their children [39,40].

The adaptation of the family member to institutionalization is related to various factors, one of them is the prior decision-making process about admission. Dellasega and Mastrian [41] reported how the institutionalization of the elderly conflicted with the family’s vision of themselves as the ideal caregivers. This trade-off leads to emotional confusion, ambivalence, and a need to redefine the role that the family was playing. Kwon and Tae [42] suggest that in Western countries the decision tends to be made alone by the main caregiver or the elderly person, in contrast to what happens in Eastern nations, in which it is a family matter in which all members, especially the children, take part.

Dung et al. [43] recently shared the results of a study that included 900 older people living in nine nursing homes in three regions of Vietnam, as well as managers and staff in the homes. The results showed that more than half of the older respondents wanted to live in nursing homes. They chose to spend their lives in these facilities because they were homeless, had no children, or had lost their family (through divorce, death, or missing in wars). The rest preferred to live with their family because they considered family care to be irreplaceable as they grew older. For the authors, these results indicate that a good tradition of Vietnamese culture regarding the elderly and the filial piety that is manifested in their care is disappearing.

Furthermore, Deimling et al. [44], reported on different decision-making patterns between Afro-American and European-American families. The former were less likely to include the elderly when making decisions, as well as to consult with a health professional; on the other hand, in European-American families, the elderly were central participants in decisions about their care and physicians played an important role as guidance.

Important differences were also found in the reasons for admission, potentially associated with the socioeconomic levels of family members, such as the need to work, was a more influential reason for the decision to admit seniors in the case of LINH, whereas in HINH, it was the senior’s worsening health.

In line with other authors’ findings [39,40], in Colombian LINH, there was a higher percentage of men than in HINH. Osorio and Salinas [45] attributed this to the fact that family members expressed the preference to take care of their mother at home because they were more devoted to their home, whereas fathers were taken to the nursing home, presenting cases where they do not want to see their father, limiting them to making the monthly payment.

However, in spite of the differences outlined, our study also reveals similarities between family members of the senior from LINH and HINH, highlighting the complexity of the social networks maintained by senior residents, regardless of the type of center. Therefore, in the total sample, the daughters who served the role of main caregivers were most common, but there were also other family members and even neighbors and friends who continued to take care of the institutionalized senior. According to data from the National Study of Health and Aging (SABE) [46,47], 85.6% of Colombian older adults reported living with other individuals with different degrees of kinship by blood and marriage; as well as with other people (e.g., tenants, companions, caregivers, and workers). Our results are also consistent with what has been reported regarding the composition of the social networks of seniors living in nursing homes [48]. Further, it points to the importance of taking these people into account in the process of seniors adapting to institutionalization in the Colombian context [49].

As reported in studies from other countries, we found that among the relatives who visit seniors, women in their 50s do so more often. Family members who work outside the home, are married, and have a high level of education also comprised the majority [16,18,19,48,49,50,51,52]. In Latin America, there has been a persistent upward trend in women’s education levels and a generalized increase in the economic activity of women, similar to the situation in Spain [53]. The more precarious the labor context, in which women are doubly overburdened by the fact that men are less involved in care, child-rearing, and housework, the more family–work balance becomes important [53].

Notably, no spouse was found in the Colombian sample. We did not find research carried out in the Ibero-American environment that provided information on the type of kinship of family members visiting seniors admitted to nursing homes in terms of frequency because, as previously stated, research has been limited and qualitative research is most common. However, in Spain, Llamazares [48] found that only 2% of the family members were spouses; other authors have confirmed that along with siblings and other relatives, spouses constituted less than 10% of the sample [23].

A possible explanation for this finding may be that some spouses “assume” that they have fewer responsibilities to their partners or that caring represents an additional task that causes important changes in their lifestyle. It may also be because partners of seniors are usually also seniors who may need care or that the death of the spouse who was in charge of the surviving senior was the trigger for admission to a nursing home [47,48]. In fact, in the Colombian older adult population, the proportion of people who live with a partner is 50.7% according to data from the SABE study [46,47]. Evidence has been found that married older people face nearly half the risk of being admitted to a nursing home [28,29,30]. We have not found previous studies in the Colombian population to support these assumptions, so it is a subject that merits research. Interestingly, in studies conducted on English-speaking populations, in contrast, it was found that the family member who comes to visit is the spouse as seen, for example, in the aforementioned study by Rosenthal and Dawson [16], which was based on the reports of the wives of seniors admitted to nursing homes.

This result may explain why in the Colombian sample, the items related to nostalgia for seniors did not have explanatory power because this component of the adaptation process was described mainly for wives of seniors admitted to nursing homes because of issues of severe dementia [16,48].

### Limitations and Future Studies

Our research has limitations that must be considered. First, as is recognized, the absence of restrictions of the EFA models prevents truly testing substantive hypotheses; therefore, the previous results of an EFA should not be considered conclusive evidence [32,33].

Another aspect that should be included in future studies is the need to deepen the analysis of content validity, combining methods based on the judgment of experts and the use of statistical methods derived from the application of the measurement instrument, as recommended by Pedrosa et al. [54], among others.

Another limitation is that the sample is relatively small for a psychometric study, although the question of sample size and composition in EFA has been an object of study by researchers for decades. The most popular recommendation is that it be a minimum of 200 cases or that there be at least 5 subjects per variable [33], but the simplicity of this requirement is currently questioned [36], particularly when it comes to instruments designed for small populations that are difficult to involve in this type of study [37].

Thus, it is worth noting the difficulties in collaborating with managers of nursing homes and family members of the seniors admitted, which have been reported by other authors [54], as well. Twelve centers were contacted, but only six were authorized, located in four cities in the Colombian Caribbean, on the northern coast of the country. Unlike what happens in other cultures [50,55,56], in Colombian culture, the idea prevails that the admission of seniors in a nursing home is synonymous with abandonment, which leads many people to reject information because of fear of stigma [45,57]. In addition, in nursing homes, the view of family caregivers as a resource or collaborator to carry out the care managed by professionals is common [45,55,58]. Therefore, on many occasions, they “censor” family members’ communication.

An added difficulty to the limitation of the sample size consists of determining the differences that the LINH and HINH subsamples present in relation to the studied construct and also the problems in determining the extent to which the concept of family adaptation differs between family and non-family members. This seems to be a substantive element that could partially explain the differences obtained in the samples from Colombia and Spain. In any case, this situation responds to the reality of both countries, but it is necessary to be able to work with a larger sample size that allows us to establish these implications.

Other limitations would be those of studies with limited financial resources, in which close supervision of the procedures for data collection is not possible, which may lead to an increase in the risk of external variables that are not initially considered in research and have explanatory power, such as the expertise of the interviewers or how the interviewees may have interpreted the questions.

Thus, this study generated numerous questions that can be pursued in future studies. Although the CAFIAR-15 was not appropriate for the evaluation of Colombian family members, we found that the idea of having an instrument that promotes the quantity and quality of healthcare and investigative work in this important area should not be dismissed.

As we have already pointed out, research on the families of the elderly living in geriatric homes is precarious. Recently, Pritty et al. [59] published a systematic review on the adaptation of the family member to the admission of the elderly, finding that the studies originated mainly in four countries: USA, Sweden, Australia, and Canada. All the investigations had in common that the strategy for obtaining the data was qualitative, be it semi-structured interviews or focus groups. In the Ibero-American context, research on elderly relatives in geriatric homes has been more limited, and it has also been carried out basically with qualitative strategies [9]. The lack of studies in our environment may indicate that there is less sensitivity and interest on the part of researchers in the subject. This could be due to the fact that, in our countries, the responsibility of health systems and the state in the care of the elderly is less recognized, and there is more emphasis on family responsibility than on social responsibility [9,60,61,62,63,64,65], unlike other cultures, such as North American or English [50,55,65]. Thus, in our environment, institutional care services are usually organized around the elderly and on many occasions, the family is considered as another resource. Therefore, the interest is focused on maintaining the family in its role as caregivers, forgetting that the admission of the elderly person to a geriatric home is an event in which the whole family is involved and has repercussions on their dynamics and well-being.

However, the limited research on this topic does not rule out its importance, on the contrary, as we have already commented, it has been shown that admission to a nursing home can be one of the most important events in the life of an elderly person and their family; and that on many occasions, the family continues to be involved in the care of their dependent family member, wanting to actively participate in it. The importance of caring for the family of the elderly living in a geriatric home has increased dramatically as a result of the impact of the COVID-19 pandemic, as it is beginning to be recognized by different authors [66,67,68,69,70]

In the near future, studying family members’ adaptations to a senior’s admission to a nursing home will become relevant because of the impact of the COVID-19 pandemic on the mortality of residents at these institutions and the limitations imposed on communication and personal encounters between seniors and their families [4,8,66,69,70].

## 5. Conclusions

Cultural differences and differences between the LINH and HINH may be the basis for the flaws detected in the conceptual validity of the CAFIAR-15 in the Colombian sample. This motivates us to continue research on the items and dimensions that should truly be considered markers of the family member’s adaptation to a senior’s admission to a nursing home construct, with value in the Colombian population.

This purpose raises the need to collect more information that allows us to clearly establish the behavior of the instrument’s structure in both contexts, thus establishing the very nature of the construct studied both among family members of elderly admitted, and among informal family and non-family caregivers.

## Figures and Tables

**Table 1:** Sociodemographic information.

	Nursing Homes (HINH)		Geriatric Homes (LINH)	
	N	%	N	%
Sex				
Female	53	75.7	44	63.8
Male	17	24.3	25	36.2
Marital Status				
Married	40	59.7	21	30.9
Single	15	22.4	12	17.6
Informal union			20	29.4
Widowed	7	10.4	9	13.2
Divorced/separated	5	7.5	6	8.9
Employment situation				
Domestic labor	10	14.3	20	29.4
Part time job	19	27.2	26	38.2
Full time job	25	35.7	7	10.3
Student	1	1.4		
Unemployed	1	1.4	8	11.8
Retired	7	10	5	7.4
Other	7	10	2	2.9
Education				
Uneducated			2	2.9
Primary	2	2.9	9	13.2
Secondary	7	10.3	33	48.5
Higher	59	86.8	24	35.4

**Table 2:** CAFIAR-15 items descriptive statistics.

Item	N Valid N Lost		Mean	Mode	Asymmetry	Asymmetry Tip Error	Kurtosis	Kurtosis Tip Error	Minimum	Maximum	Corrected Total Element Correlation
I feel guilty about my family member being admitted to a nursing home.	138	1	1.77	1	0.691	0.206	−1.054	0.410	1	4	0.076
I feel like I should not have left my family member at the nursing home.	138	1	1.98	1	0.575	0.206	−0.642	0.410	1	5	0.090
I miss my family member.	138	1	3.18	3	−0.064	0.206	−0.882	0.410	1	5	0.074
I feel like I have more time for myself.	138	1	2.86	3	0.198	0.206	−0.803	0.410	1	5	0.403
I am relieved.	138	1	3.20	3	0.023	0.206	−0.560	0.410	1	5	0.271
I am concerned that the nursing home staff will not take good care of my family member.	139	0	2.32	1	0.594	0.206	−0.815	0.408	1	5	0.111
I have enjoyed new activities.	138	1	2.80	3	0.144	0.206	−0.856	0.410	1	5	0.337
I get more done on a daily basis.	137	2	3.28	3	−0.113	0.207	−0.926	0.411	1	5	0.109
I want my family member to come home.	139	0	2.96	3	0.013	0.206	−0.886	0.408	1	5	−0.118
I think my family member’s health is going to get worse in the short term.	136	3	2.56	1	0.372	0.208	−1.118	0.413	1	5	0.045
I feel that another person is capable of caring for my family member.	137	2	2.01	1	1.011	0.207	−0.269	0.411	1	5	0.002
I have enjoyed getting back to activities that I was previously unable to do.	138	1	2.91	3	0.170	0.206	−0.735	0.410	1	5	0.268
I think my family member’s health is going to deteriorate because of being in a nursing home.	138	1	1.96	1	0.903	0.206	−0.370	0.410	1	5	−0.160
I have adapted to the change that this situation caused in my family’s relationship.	138	1	4.23	5	−1.080	0.206	0.082	0.410	1	5	0.272
I have accepted that my family member is in the nursing home.	138	1	4.25	5	−1.197	0.206	0.380	0.410	1	5	0.303

**Table 3:** Configuration matrix.

Items	Factor		
	1	2	3
I feel like I have more time for myself.	0.617	0.348	
I have enjoyed getting back to activities that I was previously unable to do.	0.586		
I have enjoyed new activities.	0.581		
I get more done on a daily basis.	0.566		
I am relieved.	0.446		
I want my family member to come home.	−0.343		
I am concerned that the nursing home staff will not take good care of my family member.	−0.320		
I think my family member’s health is going to get worse in the short term.	−0.313		
I miss my family member.			
I feel that another person cannot take care of my family member.			
I have accepted that my family member is in the nursing home.		0.885	
I have adapted to the change that this situation caused in my relationship with my family member.		0.838	
I feel like I should not have left my family member at the nursing home.			0.778
I feel guilty about my family member being admitted to the nursing home.			0.683
I think my family member’s health is going to deteriorate because of being in a nursing home.			0.420

Extraction method: principal axis factoring; rotation method: oblimin with Kaiser normalization.
